# Supplementary material for: Disclosing the Interactome of Leukemogenic NUP98-HOXA9 and SET-NUP214 Fusion Proteins Using a Proteomic Approach
Source: Cells. 2020 Jul 10;9(7):1666. doi: 10.3390/cells9071666 (PMC7407662; doi:10.3390/cells9071666)
Supplement: Supplementary file 1 [file cells-09-01666-s001.pdf]

Supplementary Information

	Control	Bait proteins	
Hits	BirA^{R118G}	NHA9-BioID	SN214-BioID
BirA^{R118G}*	10	8	5
A*	15	2	15

*Numbers represesnt LFQ values of each hit determined by MS in each bait condition

1. **Internal normalization of protein A (LFQi)**

LFQi ($A_{BirA(R118G)}$) = 15/10 = 1,5

LFQi ($A_{NHA9\text{-}BioID}$) = 2/8 = 0,25

LFQi ($A_{SN214\text{-}BioID}$) = 15/5 =3

2. External normalization of protein A (LFQe): bait LFQi$_A$ / control LFQi$_A$

LFQe ($A_{BirA(R118G)}$) = 1,5/1,5 = 1

LFQe ($A_{NHA9\text{-}BioID}$) = 0,25/1,5 = 0,167

LFQe ($A_{SN214\text{-}BioID}$) = 3/1,5 = 2

Log$_2$ Transformed values (Fold Change, F.C.)

Log$_2$ ($A_{BirA(R118G)}$) = 0 (No change)

Log$_2$ ($A_{NHA9\text{-}BioID}$) = -2,58 (less abundant in NHA9-BioID than in control BioID)

Log$_2$ ($A_{SN214\text{-}BioID}$) = 1 (twice more abundant in SN214-BioID than in control BioID)

Figure S1: Normalization strategy of BioID results. Normalization of protein MS-Label Free Quantification (LFQ) values between NHA9-BioID and BirA^{R118G} and SN214-BioID and BirA^{R118G}. A two-step normalization approach was used to account for the differences in protein expression between the BioID fusion proteins and the control BirA^{R118G}. **1.** *Internal normalization* (LFQ$_i$): for each condition, the ratio between the LFQ value of each protein (protein *A*) and the LFQ value of the BirA^{R118G} *within* the same condition (i.e. the BirA portion from the respective BioID fusion protein). **2.** *External normalization* (LFQ$_e$): the LFQ$_i$ value of each protein (protein *A*) in NHA9-BioID or SN214-BioID was divided by the corresponding LFQ$_i$ value in the control condition (BirA). The results are expressed as a fold-change, which corresponds to log2.

Function	Group Genes
cytoskeletal response to stress	ABLIM1 | ACTB | BAG3 | ELMO3 | PTK2 | SEPT11
deactivation of the beta-catenin transactivating complex	BCL9L | SOX9 | TLE3
estrogen-dependent gene expression	CALM3 | CDC23 | DVL1 | JUN | NCOA3 | POLR2E | SCRIB | TLE3 | TNRC6A | TNRC6B
MAPK6/MAPK4 signaling	IPO8 | JUN | NCOA3 | NUP58 | POLR2E | PSMB6 | RAE1 | TLE3 | TNRC6A | TNRC6B
mitochondrial protein import	BCS1L | MTX1 | SLC25A13 | TOMM7
TP53 Regulates Transcription of DNA Repair Genes	CTDP1 | JUN | POLR2E
basal RNA polymerase II transcription machinery binding	CTDP1 | NCOA3 | SOX9
RNA processing	BAG3 | CALM3 | CLPB | EIF2A | GNPDA1 | IPO8 | MAPT | NCOA3 | NUP58 | PIAS2 | POLR2E | RAE1 | SLBP | SLC25A13 | SLU7 | TNRC6A | TNRC6B
inner ear morphogenesis	CEP290 | DVL1 | SCRIB | SOX9 | TFAP2A | TRIOBP | KIF3A | SCRIB | TRIOBP
mitochondrial respiratory chain complex assembly	BCS1L | LYRM7 | NDUFA2 | NDUFA3
mitotic metaphase plate congression	CDC23 | CHMP6 | ZW10
negative regulation of EGFR signaling pathway	CHMP6 | EPS15 | SLBP
neural nucleus development	ACTB | AKAP9 | CALM3 | GNAQ | KIF3A | SCRIB | ZNF148
cell proliferation and mitophagy	JUN | MEF2D | MFN2 | TOMM7

Figure S2: Clustered Pathway Analysis of NUP98-HOXA9-BioID proximal interactors. Functionally grouped NHA9-BioID interactors. Statistical analysis was performed with the Cytoscape plugin ClueGo (v2.5.5) using hypergeometric test and the following parameters: p <0.01, kappa-score (k) = 0.4; (min/%) genes = 3/4%, GO tree levels: 3-11. Ontology databases: GOBP, GOCC, and GOMF, Reactome Pathways and KEGG.

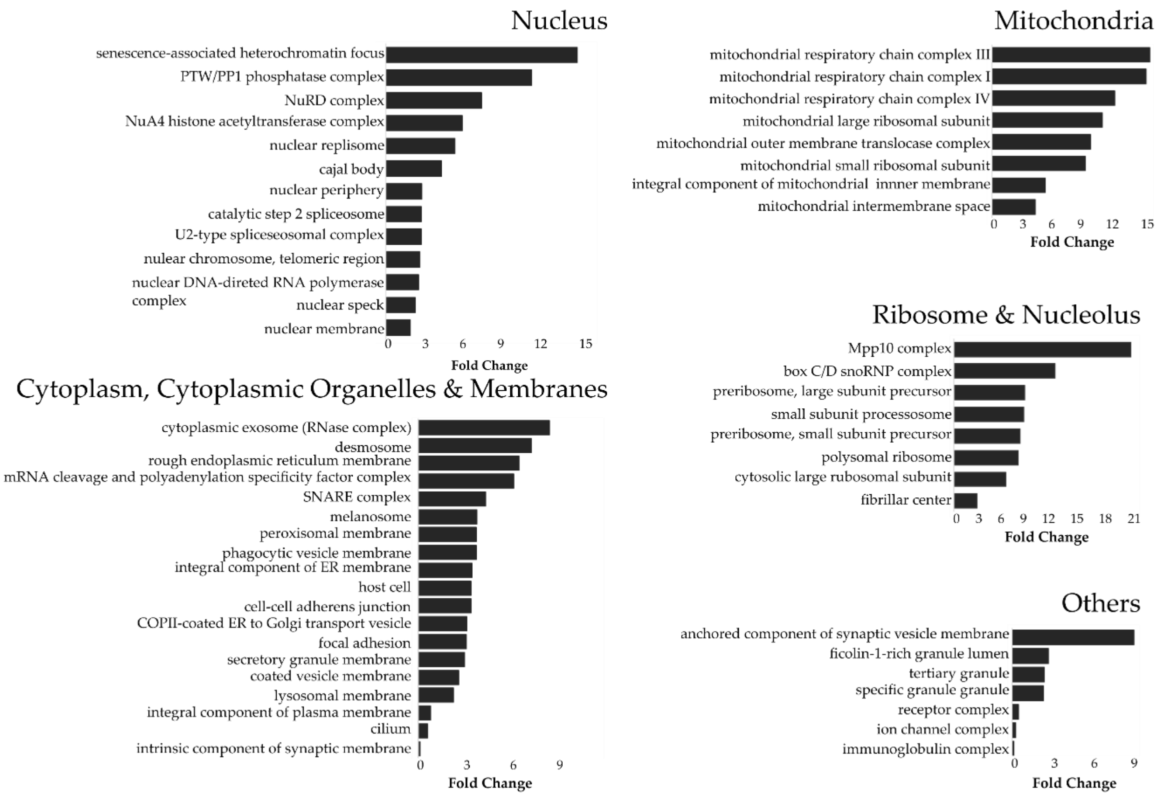

Figure S3: Most represented cellular compartments (GOCC) among SN214-BioID proximal interactors. Statistical analysis of the overrepresented proteins in the SN214-BioID fraction (total 1125 proteins) with PANTHER classification online software (v14.1) using Fisher's exact test. Results are displayed for FDR p <0.05.

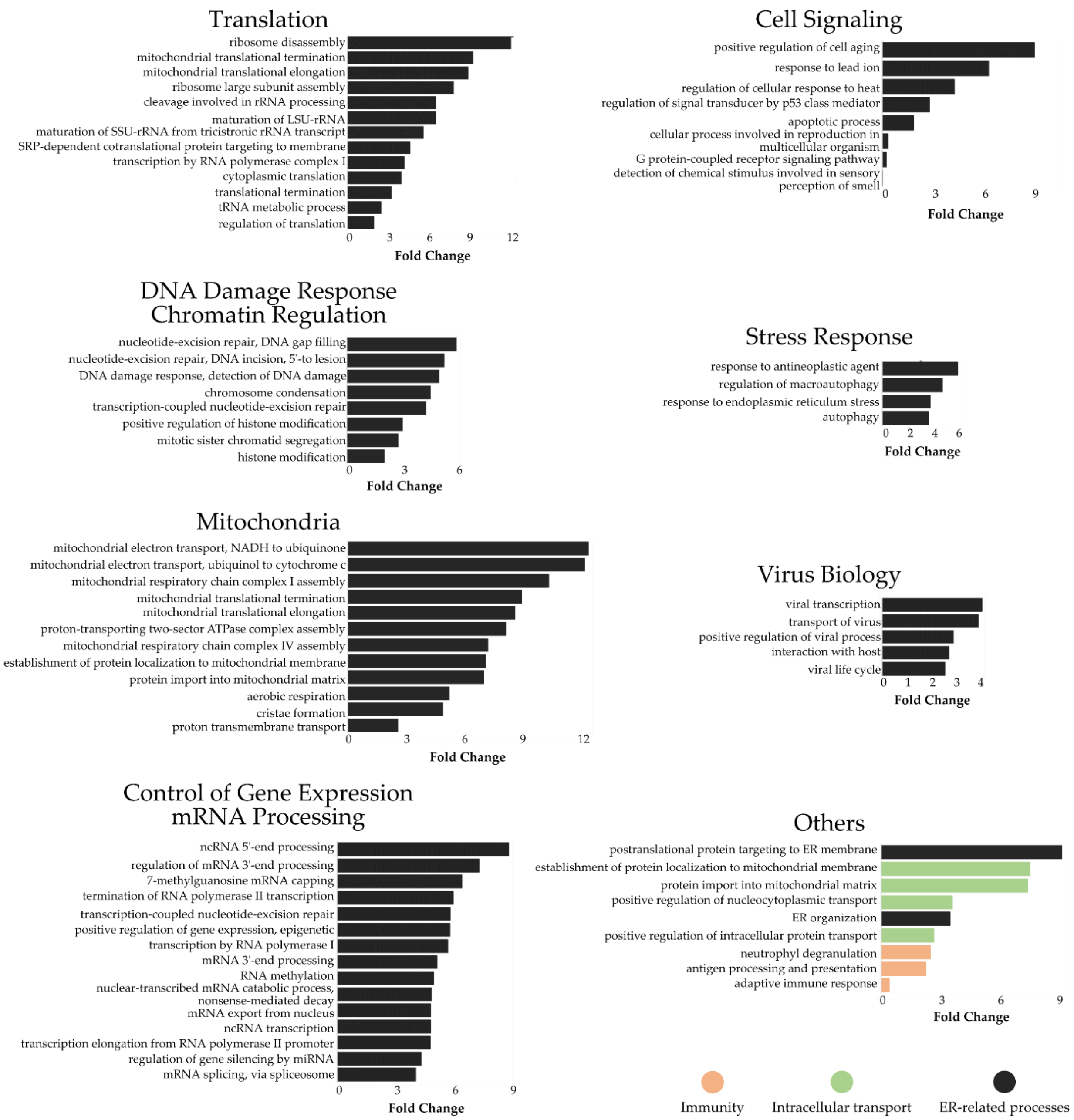

Figure S4: Most represented biological processes (GOBP) among SN214-BioID proximal interactors. Statistical analysis of the overrepresented proteins in the SN214-BioID fraction (total 1125 proteins) with PANTHER classification online software (v14.1) using Fisher's exact test. Results are displayed for FDR p <0.05.

A.

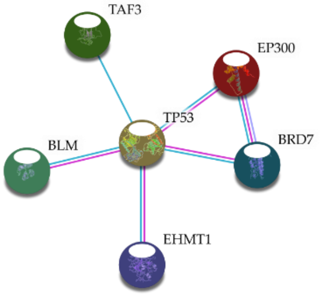

Protein name	Gene	SN214-BioID (F.C.)
Bloom syndrome protein	BLM	29,42
Histone acetyltransferase p300	EP300	26,67
Transcription initiation factor TFIID subunit 3	TAF3	1,72
Histone-lysine N-methyltransferase EHMT1	EHMT1	26,68
Bromodomain-containing protein 7	BRD7	27,01

B.

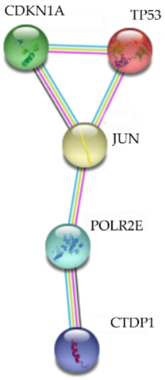

Protein name	Gene	NHA9-BioID (F.C.)
Transcription factor AP-1	JUN	28,64
DNA-directed RNA polymerase II subunit E	POLR2E	26,06
RNA polymerase II subunit A C-terminal domain phosphatase	CTDP1	27.50

Figure S5: **Network of direct p53 binding proteins in SN214-BioID and NHA9-BioID proximal interactors**. STRING (https://string-db.org/) network of direct p53 binding proteins found in the pool of **A.** SN214-BioID and **B.** NHA9-BioID proximal interactors, and the corresponding fold change values relative to control BirAR118G. TP53 and CDKN1A were included to illustrate protein-protein interactions.

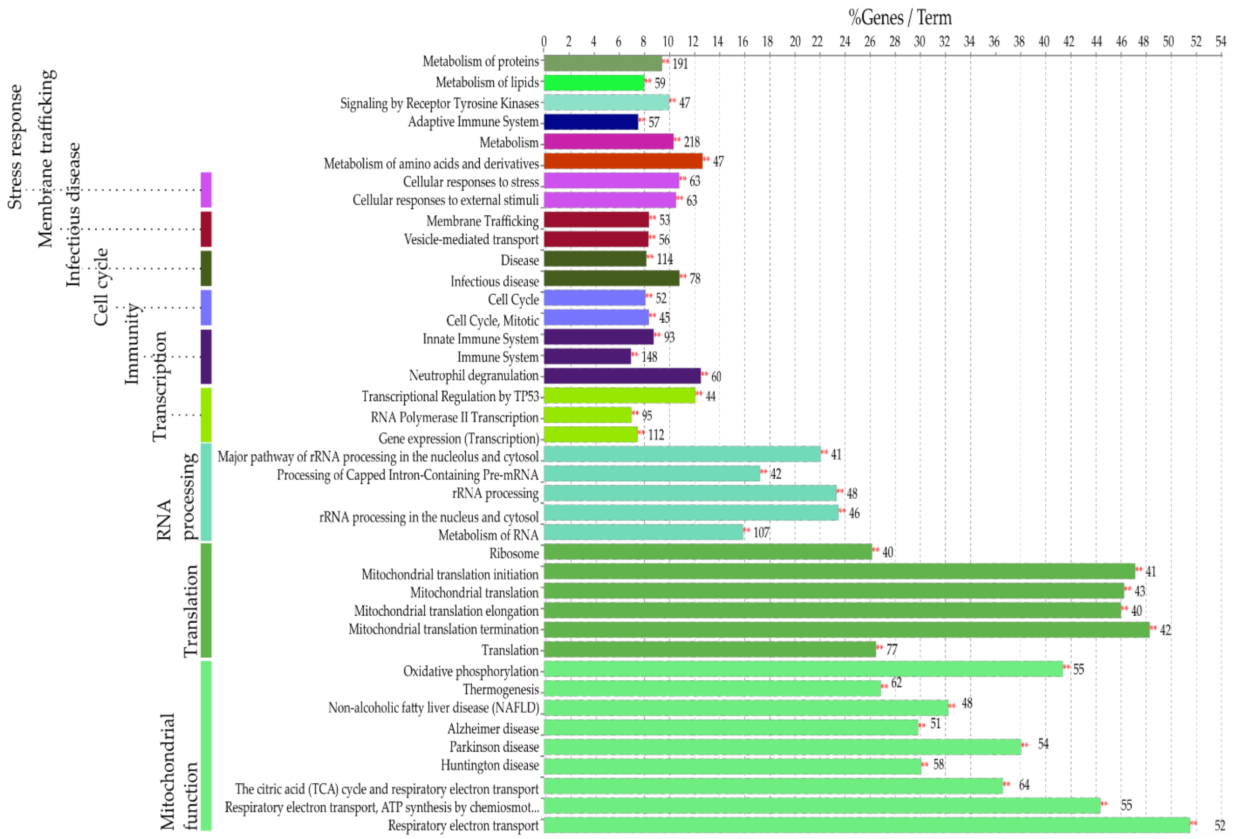

Figure S6: Clustered Pathway Analysis of SET-NUP2147-BioID proximal interactors. Graphic representation of functional groups. The of number of genes within each group is shown after each bar. The X axis corresponds to the percentage of genes/term that is present in the protein set, by comparison to the reference organism (*Homo sapiens* genome). Statistical analysis was performed with the Cytoscape plugin ClueGo (v2.5.5) using the following parameters: p <0.01, kappa-score (k) = 0.5; (min/%) genes = 40/4%, GO tree levels: 13-15. Ontology databases: GOBP, GOCC, and GOMF, Reactome Pathways and KEGG.

Table S1
List of biotinylated proteins found exclusively in the biotinylated fraction of $BirA^{R118G}$

Protein name	UniPROT ID	Gene	Cellular localization*
Ubiquitin family domain containing 1	O14562	UBFD1	N/A
Sperm associated antigen 9	O60271	SPAG9	Lysosome
Methyltransferase like 18	O95568	METTL18	n/a
Polypyrimidine tract binding protein 3	O95758	PTBP3	Nucleus
Thioredoxin domain containing 12	O95881	TXNDC12	Endoplasmic reticulum
Interferon induced protein 35	P80217	IFI35	Nucleus
Epidermal growth factor receptor pathway substrate 8	Q12929	EPS8	Plasma membrane
Cytosolic thiouridylase subunit 2	Q2VPK5	CTU2	Cytoplasm
Poly(ADP-ribose) polymerase family member 14	Q460N5	PARP14	Nucleus
Programmed cell death 4	Q53EL6	PDCD4	Nucleus
Zinc finger protein 787	Q6DD87	ZNF787	Nucleus
Solute carrier family 27 member 1	Q6PCB7	SLC27A1	Plasma membrane
THO complex 6	Q86W42	THOC6	Nucleus
Exoribonuclease 1	Q8IV48	ERI1	Nucleus
AVL9 cell migration associated	Q8NBF6	AVL9	Endosome
Atpase family AAA domain containing 1	Q8NBU5	ATAD1	Peroxisome / Plasma membrane
Zinc finger CCHC-type containing 10	Q8TBK6	ZCCHC10	N/A
Myosin XVIIIA	Q92614	MYO18A	Golgi / Cytoskeleton
Fumarylacetoacetate hydrolase domain containing 2A	Q96GK7	FAHD2A	N/A
Tubulin folding cofactor B	Q99426	TBCB	Golgi / Cytoskeleton
WD repeat domain 26	Q9H7D7	WDR26	Nucleus / Mitochondria
Helicase, lymphoid specific	Q9NRZ9	HELLS	Nucleus
RNA binding fox-1 homolog 1	Q9NWB1	RBFOX1	Nucleus
Histone parylation factor 1	Q9NWY4	HPF1	Nucleus
Disco interacting protein 2 homolog B	Q9P265	DIP2B	Nucleus / Extracellular regions or secreted
Protein phosphatase 6 regulatory subunit 1	Q9UPN7	PPP6R1	Cytoplasm
Transportin 3	Q9Y5L0	TNPO3	Nucleus / Cytoplasm

* Cellular localization according to the UniPROT database

Table S2

List of overrepresented proteins in the biotinylated fraction of NHA9-BioID (NHA9-BioID proximal interactors) and respective fold-change relative to control BirA^{R118G}

Protein name	UniProt ID	Gene name	LFQ_e
Dedicator of cytokinesis 6	Q96HP0	DOCK6	33,239
Dynein axonemal heavy chain 5	Q8TE73	DNAH5	30,201
ATPase 13A1	Q9HD20	ATP13A1	29,197
SUB1 regulator of transcription	P53999	SUB1	29,113
Eukaryotic translation initiation factor 2A	Q9BY44	EIF2A	28,994
Jun proto-oncogene, AP-1 transcription factor subunit	P05412	JUN	28,639
Stathmin 1	P16949	STMN1	28,622
Calmodulin 3	P0DP25	CALM3	28,516
Adenylate kinase 3	Q9UIJ7	AK3	28,499
Glutamate-ammonia ligase	P15104	GLUL	28,413
**Zinc finger protein 384**	**Q8TF68**	**ZNF384**	**28,340**
RNA binding motif protein 33	Q96EV2	RBM33	28,318
Golgi reassembly stacking protein 2	Q9H8Y8	GORASP2	28,305
Epidermal growth factor receptor pathway substrate 15	P42566	EPS15	28,137
**Dishevelled segment polarity protein 1**	**O14640**	**DVL1**	**28,105**
Translational activator of cytochrome c oxidase I	Q9BSH4	TACO1	28,089
Cold inducible RNA binding protein	Q14011	CIRBP	28,027
Translocase of outer mitochondrial membrane 7	Q9P0U1	TOMM7	27,822
Mannose phosphate isomerase	P34949	MPI	27,775
Methylmalonyl-CoA mutase	P22033	MMUT	27,758
NADH:ubiquinone oxidoreductase subunit A3	O95167	NDUFA3	27,749
Cystatin A	P01040	CSTA	27,738
Coiled-coil domain containing 124	Q96CT7	CCDC124	27,695
DDB1 and CUL4 associated factor 7	P61962	DCAF7	27,593
NDRG family member 3	Q9UGV2	NDRG3	27,531
NADH:ubiquinone oxidoreductase subunit A2	O43678	NDUFA2	27,530
Prenylcysteine oxidase 1	Q9UHG3	PCYOX1	27,504
CTD phosphatase subunit 1	Q9Y5B0	CTDP1	27,500
Centrosomal protein 290	O15078	CEP290	27,448
Nuclear receptor subfamily 2 group C member 2	P49116	NR2C2	27,441
**Transcription factor AP-2 alpha**	**P05549**	**TFAP2A**	**27,398**
Gasdermin A	Q96QA5	GSDMA	27,396
Proteasome subunit beta 6	P28072	PSMB6	27,371
Microtubule associated protein tau	P10636	MAPT	27,358
**TLE family member 3, transcriptional corepressor**	**Q04726**	**TLE3**	**27,345**
TOX high mobility group box family member 3	O15405	TOX3	27,278
BRCA1/BRCA2-containing complex subunit 3	P46736	BRCC3	27,239
NAD(P)HX epimerase	Q8NCW5	NAXE	27,205
Nudix hydrolase 19	A8MXV4	NUDT19	27,195
Scribble planar cell polarity protein	Q14160	SCRIB	27,173
Metaxin 1	Q13505	MTX1	27,166
Programmed cell death 10	Q9BUL8	PDCD10	27,149
Protein tyrosine kinase 2	Q05397	PTK2	27,074
SRY-box transcription factor 9	P48436	SOX9	27,074

LYR motif containing 7	Q5U5X0	LYRM7	27,071
Syntaxin 10	O60499	STX10	27,049
Mitochondrial calcium uniporter	Q8NE86	MCU	26,975
FLYWCH family member 2	Q96CP2	FLYWCH2	26,974
Non-specific cytotoxic cell receptor protein 1 homolog (zebrafish)	Q6ZVX7	NCCRP1	26,956
Septin 11	Q9NVA2	SEPTIN11	26,933
Fatty acyl-CoA reductase 1	Q8WVX9	FAR1	26,902
Rho guanine nucleotide exchange factor 10	O15013	ARHGEF10	26,876
Hippocalcin like 1	P37235	HPCAL1	26,869
3-hydroxybutyrate dehydrogenase 1	Q02338	BDH1	26,865
BBX high mobility group box domain containing	Q8WY36	BBX	26,858
**Trinucleotide repeat containing adaptor 6A**	**Q8NDV7**	**TNRC6A**	**26,847**
ATPase H+ transporting V0 subunit d1	P61421	ATP6V0D1	26,838
Mitochondrial ribosomal protein S12	O15235	MRPS12	26,821
Acidic nuclear phosphoprotein 32 family member E	Q9BTT0	ANP32E	26,768
stem-loop binding protein	Q14493	SLBP	26,767
F-box protein 22	Q8NEZ5	FBXO22	26,705
Mitofusin 2	O95140	MFN2	26,677
F-box protein 2	Q9UK22	FBXO2	26,628
Eukaryotic translation initiation factor 2D	P41214	EIF2D	26,590
Decapping mRNA 1A	Q9NPI6	DCP1A	26,584
Activating transcription factor 3	P18847	ATF3	26,511
Nuclear factor I A	Q12857	NFIA	26,502
Kelch domain containing 4	Q8TBB5	KLHDC4	26,472
Zw10 kinetochore protein	O43264	ZW10	26,415
Pleckstrin homology like domain family B member 2	Q86SQ0	PHLDB2	26,409
Nucleoporin 58	Q9BVL2	NUP58	26,380
Protein inhibitor of activated STAT 2	O75928	PIAS2	26,358
Protein kinase C and casein kinase substrate in neurons 3	Q9UKS6	PACSIN3	26,319
A-kinase anchoring protein 9	Q99996	AKAP9	26,305
3-hydroxyacyl-CoA dehydratase 2	Q6Y1H2	HACD2	26,302
Actin binding LIM protein 1	O14639	ABLIM1	26,293
Spermatogenesis associated 5 like 1	Q9BVQ7	SPATA5L1	26,178
Cap methyltransferase 1	Q8N1G2	CMTR1	26,136
Zinc finger protein 148	Q9UQR1	ZNF148	26,107
DNA polymerase delta 3, accessory subunit	Q15054	POLD3	26,089
RNA polymerase II subunit E	P19388	POLR2E	26,062
TRIO and F-actin binding protein	Q9H2D6	TRIOBP	26,009
BCL9 like	Q86UU0	BCL9L	26,005
Novel protein	H7C1D1	AC037459.1	25,908
WD repeat and HMG-box DNA binding protein 1	O75717	WDHD1	25,897
C-X9-C motif containing 1	Q7Z7K0	CMC1	25,888
Eukaryotic translation initiation factor 4E nuclear import factor 1	Q9NRA8	EIF4ENIF1	25,885
Phosphomevalonate kinase	Q15126	PMVK	25,883
SKI proto-oncogene	P12755	SKI	25,868
FKBP prolyl isomerase 10	Q96AY3	FKBP10	25,814
Isochorismatase domain containing 1	Q96CN7	ISOC1	25,781

KIAA1671	Q9BY89	KIAA1671	25,781
Chromosome 19 open reading frame 44	Q9H6X5	C19orf44	25,731
Charged multivesicular body protein 6	Q96FZ7	CHMP6	25,731
SET domain containing 3, actin histidine methyltransferase	Q86TU7	SETD3	25,692
Cell division cycle 23	Q9UJX2	CDC23	25,614
SLU7 homolog, splicing factor	O95391	SLU7	25,612
BCS1 homolog, ubiquinol-cytochrome c reductase complex chaperone	Q9Y276	BCS1L	25,563
Amylo-alpha-1, 6-glucosidase, 4-alpha-glucanotransferase	P35573	AGL	25,521
Zinc finger CCHC-type containing 8	Q6NZY4	ZCCHC8	25,421
ClpB homolog, mitochondrial AAA ATPase chaperonin	Q9H078	CLPB	25,276
Peptidase M20 domain containing 2	Q8IYS1	PM20D2	25,252
Coiled-coil domain containing 50	Q8IVM0	CCDC50	25,249
Origin recognition complex subunit 4	O43929	ORC4	25,218
TELO2 interacting protein 1	O43156	TTI1	25,145
Glucosamine-6-phosphate deaminase 1	P46926	GNPDA1	25,143
Engulfment and cell motility 3	Q96BJ8	ELMO3	25,109
Kinesin family member 3A	Q9Y496	KIF3A	25,018
Solute carrier family 25 member 13	Q9UJS0	SLC25A13	24,847
Thiosulfate sulfurtransferase like domain containing 1	Q8NFU3	TSTD1	24,814
Nuclear factor of activated T cells 5	O94916	NFAT5	24,812
TGF-beta activated kinase 1 (MAP3K7) binding protein 2	Q9NYJ8	TAB2	24,803
NSFL1 cofactor	Q9UNZ2	NSFL1C	24,783
2-oxoglutarate and iron dependent oxygenase domain containing 3	Q6PK18	OGFOD3	24,749
Dynein light chain roadblock-type 1	Q9NP97	DYNLRB1	24,736
Lysophosphatidylcholine acyltransferase 4	Q643R3	LPCAT4	24,635
Nuclear receptor coactivator 3	Q9Y6Q9	NCOA3	24,603
SLIT-ROBO Rho GTPase activating protein 2B	P0DMP2	SRGAP2B	24,475
Signal transducer and activator of transcription 2	P52630	STAT2	24,429
BCL2 associated athanogene 3	O95817	BAG3	24,320
ER membrane protein complex subunit 1	Q8N766	EMC1	24,185
G protein subunit alpha q	P50148	GNAQ	23,202
WD repeat containing, antisense to TP73	Q9P2S5	WRAP73	22,894
Actin beta	P60709	ACTB	22,586
NUP98-HOXA9	*bait*	None	8,518
Ribonucleic acid export 1	P78406	RAE1	6,395
Nucleus accumbens associated 1	Q96RE7	NACC1	1,365
Importin 8	O15397	IPO8	1,010
Trinucleotide repeat containing adaptor 6B	Q9UPQ9	TNRC6B	0,874
Myocyte enhancer factor 2D	Q14814	MEF2D	0,841
JunB proto-oncogene, AP-1 transcription factor subunit	P17275	JUNB	0,697

Proteins highlighted in blue were exclusively detected in the NHA9-BioID pool of biotinylated proteins.

Table S3

Presence of classical nuclear export signals (NES) in NHA9-BioID proximal interactors

Protein name	Gene	F.C	NES finder 0.2	LocNES
Dedicator of cytokinesis 6	DOCK6	33,239	Y	Y
Dynein axonemal heavy chain 5	DNAH5	30,201	Y	Y
ATPase 13A1	ATP13A1	29,197	Y	Y
SUB1 regulator of transcription	SUB1	29,113	N	Y
Eukaryotic translation initiation factor 2A	EIF2A	28,994	Y	Y
Jun proto-oncogene, AP-1 transcription factor subunit	JUN	28,639	N	Y
Stathmin 1	STMN1	28,622	N	N
Calmodulin 3	CALM3	28,516	Y	Y
Adenylate kinase 3	AK3	28,499	Y	Y
Glutamate-ammonia ligase	GLUL	28,413	Y	Y
Zinc finger protein 384	ZNF384	28,34	N	Y
RNA binding motif protein 33	RBM33	28,318	Y	Y
Golgi reassembly stacking protein 2	GORASP2	28,305	Y	Y
Epidermal growth factor receptor pathway substrate 15	EPS15	28,137	Y	Y
Dishevelled segment polarity protein 1	DVL1	28,105	Y	Y
Translational activator of cytochrome c oxidase I	TACO1	28,089	Y	Y
Cold inducible RNA binding protein	CIRBP	28,027	N	Y
Translocase of outer mitochondrial membrane 7	TOMM7	27,822	Y	Y
Mannose phosphate isomerase	MPI	27,775	Y	Y
Methylmalonyl-CoA mutase	MMUT	27,758	Y	Y
NADH:ubiquinone oxidoreductase subunit A3	NDUFA3	27,749	Y	Y
Cystatin A	CSTA	27,738	N	N
Coiled-coil domain containing 124	CCDC124	27,695	Y	Y
DDB1 and CUL4 associated factor 7	DCAF7	27,593	N	Y
NDRG family member 3	NDRG3	27,531	Y	Y
NADH:ubiquinone oxidoreductase subunit A2	NDUFA2	27,53	N	Y
Prenylcysteine oxidase 1	PCYOX1	27,504	Y	Y
CTD phosphatase subunit 1	CTDP1	27,5	Y	Y
Centrosomal protein 290	CEP290	27,448	Y	Y
Nuclear receptor subfamily 2 group C member 2	NR2C2	27,441	Y	Y
Transcription factor AP-2 alpha	TFAP2A	27,398	Y	Y
Gasdermin A	GSDMA	27,396	Y	Y
Proteasome subunit beta 6	PSMB6	27,371	Y	Y
Microtubule associated protein tau	MAPT	27,358	Y	Y
TLE family member 3, transcriptional corepressor	TLE3	27,345	N	Y
TOX high mobility group box family member 3	TOX3	27,278	N	Y
BRCA1/BRCA2-containing complex subunit 3	BRCC3	27,239	N	Y
NAD(P)HX epimerase	NAXE	27,205	Y	Y
Nudix hydrolase 19	NUDT19	27,195	Y	N
Scribble planar cell polarity protein	SCRIB	27,173	Y	Y
Metaxin 1	MTX1	27,166	N	Y
Programmed cell death 10	PDCD10	27,149	N	Y
Protein tyrosine kinase 2	PTK2	27,074	Y	Y

SRY-box transcription factor 9	SOX9	27,074	Y	Y
LYR motif containing 7	LYRM7	27,071	Y	Y
Syntaxin 10	STX10	27,049	Y	Y
Mitochondrial calcium uniporter	MCU	26,975	Y	Y
FLYWCH family member 2	FLYWCH2	26,974	Y	N
Non-specific cytotoxic cell receptor protein 1 homolog (zebrafish)	NCCRP1	26,956	Y	N
Septin 11	SEPTIN11	26,933	Y	Y
Fatty acyl-CoA reductase 1	FAR1	26,902	Y	Y
Rho guanine nucleotide exchange factor 10	ARHGEF10	26,876	Y	Y
Hippocalcin like 1	HPCAL1	26,869	Y	Y
3-hydroxybutyrate dehydrogenase 1	BDH1	26,865	Y	Y
BBX high mobility group box domain containing	BBX	26,858	Y	Y
Trinucleotide repeat containing adaptor 6A	TNRC6A	26,847	Y	Y
ATPase H+ transporting V0 subunit d1	ATP6V0D1	26,838	Y	Y
Mitochondrial ribosomal protein S12	MRPS12	26,821	N	Y
Acidic nuclear phosphoprotein 32 family member E	ANP32E	26,768	Y	Y
stem-loop binding protein	SLBP	26,767	N	N
F-box protein 22	FBXO22	26,705	Y	Y
Mitofusin 2	MFN2	26,677	Y	Y
F-box protein 2	FBXO2	26,628	N	Y
Eukaryotic translation initiation factor 2D	EIF2D	26,59	N	Y
Decapping mRNA 1A	DCP1A	26,584	Y	Y
Activating transcription factor 3	ATF3	26,511	N	Y
Nuclear factor I A	NFIA	26,502	Y	Y
Kelch domain containing 4	KLHDC4	26,472	Y	Y
Zw10 kinetochore protein	ZW10	26,415	Y	Y
Pleckstrin homology like domain family B member 2	PHLDB2	26,409	Y	Y
Nucleoporin 58	NUPL1	26,38	Y	Y
Protein inhibitor of activated STAT 2	PIAS2	26,358	Y	Y
Protein kinase C and casein kinase substrate in neurons 3	PACSIN3	26,319	Y	Y
A-kinase anchoring protein 9	AKAP9	26,305	Y	Y
3-hydroxyacyl-CoA dehydratase 2	HACD2	26,302	Y	Y
Actin binding LIM protein 1	ABLIM1	26,293	Y	Y
Spermatogenesis associated 5 like 1	SPATA5L1	26,178	Y	Y
Cap methyltransferase 1	CMTR1	26,136	Y	Y
Zinc finger protein 148	ZNF148	26,107	N	Y
DNA polymerase delta 3, accessory subunit	POLD3	26,089	Y	N
RNA polymerase II subunit E	POLR2E	26,062	N	N
TRIO and F-actin binding protein	TRIOBP	26,009	Y	Y
BCL9 like	BCL9L	26,005	N	Y
Novel protein	AC037459.1	25,908	Y	Y
WD repeat and HMG-box DNA binding protein 1	WDHD1	25,897	Y	Y
C-X9-C motif containing 1	CMC1	25,888	N	Y

Eukaryotic translation initiation factor 4E nuclear import factor 1	EIF4ENIF1	25,885	Y	Y
Phosphomevalonate kinase	PMVK	25,883	N	Y
SKI proto-oncogene	SKI	25,868	Y	Y
FKBP prolyl isomerase 10	FKBP10	25,814	Y	Y
Isochorismatase domain containing 1	ISOC1	25,781	Y	Y
KIAA1671	KIAA1671	25,781	Y	Y
Chromosome 19 open reading frame 44	C19orf44	25,731	Y	Y
Charged multivesicular body protein 6	CHMP6	25,731	Y	Y
SET domain containing 3, actin histidine methyltransferase	SETD3	25,692	Y	Y
Cell division cycle 23	CDC23	25,614	Y	Y
SLU7 homolog, splicing factor	SLU7	25,612	Y	Y
BCS1 homolog, ubiquinol-cytochrome c reductase complex chaperone	BCS1L	25,563	Y	Y
Amylo-alpha-1, 6-glucosidase, 4-alpha-glucanotransferase	AGL	25,521	Y	Y
Zinc finger CCHC-type containing 8	ZCCHC8	25,421	Y	Y
ClpB homolog, mitochondrial AAA ATPase chaperonin	CLPB	25,276	Y	Y
Peptidase M20 domain containing 2	PM20D2	25,252	Y	Y
Coiled-coil domain containing 50	CCDC50	25,249	Y	Y
Origin recognition complex subunit 4	ORC4	25,218	Y	Y
TELO2 interacting protein 1	TTI1	25,145	Y	Y
Glucosamine-6-phosphate deaminase 1	GNPDA1	25,143	Y	Y
Engulfment and cell motility 3	ELMO3	25,109	Y	Y
Kinesin family member 3A	KIF3A	25,018	Y	Y
Solute carrier family 25 member 13	SLC25A13	24,847	Y	Y
Thiosulfate sulfurtransferase like domain containing 1	TSTD1	24,814	Y	Y
Nuclear factor of activated T cells 5	NFAT5	24,812	Y	Y
TGF-beta activated kinase 1 (MAP3K7) binding protein 2	TAB2	24,803	Y	Y
NSFL1 cofactor	NSFL1C	24,783	N	Y
2-oxoglutarate and iron dependent oxygenase domain containing 3	OGFOD3	24,749	N	Y
Dynein light chain roadblock-type 1	DYNLRB1	24,736	N	Y
Lysophosphatidylcholine acyltransferase 4	LPCAT4	24,635	Y	Y
Nuclear receptor coactivator 3	NCOA3	24,603	Y	Y
SLIT-ROBO Rho GTPase activating protein 2B	SRGAP2B	24,475	Y	Y
Signal transducer and activator of transcription 2	STAT2	24,429	Y	Y
BCL2 associated athanogene 3	BAG3	24,32	N	Y
ER membrane protein complex subunit 1	EMC1	24,185	Y	Y
G protein subunit alpha q	GNAQ	23,202	Y	Y
WD repeat containing, antisense to TP73	WRAP73	22,894	Y	Y
Actin beta	ACTB	22,586	Y	Y
Ribonucleic acid export 1	RAE1	6,395	Y	N
Nucleus accumbens associated 1	NACC1	1,365	N	Y
Importin 8	IPO8	1,01	Y	Y
Trinucleotide repeat containing adaptor 6B	TNRC6B	0,874	Y	Y

Myocyte enhancer factor 2D	MEF2D	0,841	Y	Y
JunB proto-oncogene, AP-1 transcription factor subunit	JUNB	0,697	N	Y

F.C. – Fold Change; Y - presence of at least one potential NES peptide sequence; N - absence of potential NES peptide sequences

Table S5
List of functional groups with corresponding genes of SN214-BioID proximal interactors generated by the ClueGO Cytoscape plugin

Functional Cluster	Genes
Adaptive Immune System	ACTR1B ǀ AHCYL1 ǀ ANAPC7 ǀ AP1S1 ǀ AP2A2 ǀ AP2M1 ǀ B2M ǀ BLMH ǀ CALM3 ǀ CD81 ǀ CD99 ǀ CDC27 ǀ CDH1 ǀ CHUK ǀ CSK ǀ CTSA ǀ CTSB ǀ CUL5 ǀ CYBA ǀ DTX3L ǀ DYNC1I2 ǀ DYNLL2 ǀ FBXO22 ǀ GAN ǀ HERC4 ǀ HLA-G ǀ KIF23 ǀ LRSAM1 ǀ LYN ǀ NEDD4 ǀ NFKB1 ǀ NRAS ǀ PIK3CB ǀ PIK3R2 ǀ PPP2CA ǀ PSMB4 ǀ PSMB7 ǀ PVR ǀ RAC1 ǀ RACGAP1 ǀ RAP1A ǀ RBBP6 ǀ RBX1 ǀ RNF126 ǀ SEC13 ǀ SEC22B ǀ SEC61A1 ǀ SEC61B ǀ SNAP23 ǀ TAB2 ǀ TAP2 ǀ TRIM21 ǀ UBA3 ǀ UBE2A ǀ UBE2L6 ǀ UBE2V2 ǀ UBE4A
Cell Cycle	AKAP9 ǀ ANAPC7 ǀ ATRIP ǀ BABAM2 ǀ BLM ǀ CDC27 ǀ CENPC ǀ CEP290 ǀ CLASP2 ǀ CSNK1D ǀ DHFR ǀ DYNC1I2 ǀ DYNLL2 ǀ EP300 ǀ GORASP2 ǀ H2AFV ǀ HIST2H2AC ǀ KIF23 ǀ LCMT1 ǀ LEMD3 ǀ LIG1 ǀ LMNA ǀ LYN ǀ MAD2L1 ǀ NCAPG ǀ NEK9 ǀ NHP2 ǀ NIPBL ǀ NUP153 ǀ NUP58 ǀ NUP62 ǀ PHLDA1 ǀ POLD1 ǀ POLD2 ǀ POLD3 ǀ PPP1CB ǀ PPP1R12A ǀ PPP2CA ǀ PSMB4 ǀ PSMB7 ǀ RAE1 ǀ RANBP2 ǀ RBBP7 ǀ RBX1 ǀ RFC1 ǀ RPA2 ǀ RPA3 ǀ SEC13 ǀ STAG2 ǀ SUN2 ǀ TPX2 ǀ UBE2V2
Stress response	AKT1S1 ǀ ANAPC7 ǀ ASF1A ǀ ATP6V0D1 ǀ ATP6V1E1 ǀ ATP6V1G1 ǀ CAMK2G ǀ CAT ǀ CBX4 ǀ CDC27 ǀ CYBA ǀ DNAJB6 ǀ DYNC1I2 ǀ DYNLL2 ǀ EHMT1 ǀ EP300 ǀ H2AFV ǀ HIGD1A ǀ HIST1H1C ǀ HIST1H1E ǀ HIST2H2AC ǀ HMGA1 ǀ HMGA2 ǀ HSPA6 ǀ LAMTOR3 ǀ LIMD1 ǀ NFKB1 ǀ NUP153 ǀ NUP58 ǀ NUP62 ǀ PHC2 ǀ PSMB4 ǀ PSMB7 ǀ RAE1 ǀ RANBP2 ǀ RBBP7 ǀ RBX1 ǀ RNF2 ǀ RPA2 ǀ RPA3 ǀ RPL13 ǀ RPL14 ǀ RPL18 ǀ RPL22L1 ǀ RPL23A ǀ RPL24 ǀ RPL26L1 ǀ RPL29 ǀ RPL31 ǀ RPL32 ǀ RPL34 ǀ RPL35 ǀ RPL36A ǀ RPL6 ǀ RPL7A ǀ RPLP1 ǀ RPS19BP1 ǀ RPS24 ǀ RPS26 ǀ RPTOR ǀ SEC13 ǀ TNRC6B ǀ TXNRD1
Infectious disease	ACACA ǀ AGK ǀ AKAP9 ǀ AKT1S1 ǀ AP1S1 ǀ AP2A2 ǀ AP2M1 ǀ B2M ǀ BAD ǀ BSG ǀ C1QBP ǀ CALM3 ǀ CAMK2G ǀ CAST ǀ CCNT1 ǀ CD9 ǀ CDH1 ǀ CHMP2A ǀ CHMP4B ǀ CHUK ǀ CPSF4 ǀ CREB1 ǀ CSK ǀ CTSA ǀ CUL5 ǀ DERL2 ǀ DPM3 ǀ DYNC1I2 ǀ DYNLL2 ǀ ELL ǀ EP300 ǀ EPS15 ǀ ERCC2 ǀ EREG ǀ GBE1 ǀ GRSF1 ǀ GTF2E2 ǀ HIST2H2AB ǀ HIST2H2AC ǀ HMGA1 ǀ ISG15 ǀ LIG1 ǀ LMNA ǀ MECP2 ǀ MPI ǀ MUCL1 ǀ MVB12A ǀ NCBP2 ǀ NCOR2 ǀ NELFE ǀ NFKB1 ǀ NMT1 ǀ NRAS ǀ NUP153 ǀ NUP58 ǀ NUP62 ǀ PABPN1 ǀ PAH ǀ PEBP1 ǀ PHB ǀ PIK3CB ǀ PIK3R2 ǀ PML ǀ POLR2E ǀ POLR2G ǀ POLR2I ǀ POLR2L ǀ PPP2CA ǀ PSMB4 ǀ PSMB7 ǀ RAB5A ǀ RAC1 ǀ RAC2 ǀ RAE1 ǀ RANBP2 ǀ RAP1A ǀ RBBP7 ǀ RBPJ ǀ RBX1 ǀ RCC1 ǀ RPL13 ǀ RPL14 ǀ RPL18 ǀ RPL22L1 ǀ RPL23A ǀ RPL24 ǀ RPL26L1 ǀ RPL29 ǀ RPL31 ǀ RPL32 ǀ RPL34 ǀ RPL35 ǀ RPL36A ǀ RPL6 ǀ RPL7A ǀ RPLP1 ǀ RPS24 ǀ RPS26 ǀ SDC4 ǀ SEC13 ǀ SFPQ ǀ SLC16A1 ǀ SLC25A4 ǀ SLC27A4 ǀ SLC2A1 ǀ SMAD3 ǀ SYT1 ǀ TAF2 ǀ TAF3 ǀ TBP ǀ TXNRD1 ǀ VPS25 ǀ VTA1 ǀ XRCC4
Metabolism	AASDHPPT ǀ ABCC1 ǀ ACACA ǀ ACACB ǀ ACAD8 ǀ ACADVL ǀ ACAT2 ǀ ACOT13 ǀ ACSF3 ǀ AGK ǀ AGPAT5 ǀ AHCYL1 ǀ AKR7A2 ǀ ALDH2 ǀ ALDH4A1 ǀ APOC3 ǀ ARG1 ǀ ATP5MG ǀ ATP5PF ǀ ATP6 ǀ AUH ǀ AZIN2 ǀ B4GALT5 ǀ BSG ǀ CALM3 ǀ CDIPT ǀ CDS2 ǀ CERS2 ǀ CHPF ǀ COASY ǀ COL4A3BP ǀ COX1 ǀ COX2 ǀ COX20 ǀ COX4I1 ǀ COX5B ǀ COX6C ǀ COX7A2L ǀ CPOX ǀ CTSA ǀ CYB5A ǀ CYB5R3 ǀ CYC1 ǀ CYP2S1 ǀ DCTD ǀ DGAT1 ǀ DHCR7 ǀ DHFR ǀ DHRS7B ǀ DLAT ǀ DLST ǀ ELOVL1 ǀ ELOVL5 ǀ ELOVL7 ǀ EP300 ǀ ERCC2 ǀ ESD ǀ ESYT2 ǀ ETFB ǀ FABP5 ǀ GAPDH ǀ GBE1 ǀ GCDH ǀ GLRX ǀ GNA11 ǀ GNG12 ǀ GOT1 ǀ GPAT3 ǀ GPD1L ǀ GSTK1 ǀ HACD2 ǀ HELZ2 ǀ HSD17B10 ǀ ITPA ǀ IVD ǀ KYAT3 ǀ LBR ǀ LCLAT1 ǀ LHPP ǀ LPCAT1 ǀ LPCAT3 ǀ MARCKS ǀ MBOAT7 ǀ MCAT ǀ MDH1 ǀ MDH2 ǀ ME1 ǀ MED19 ǀ MGST1 ǀ MGST2 ǀ MGST3 ǀ MLXIPL ǀ MPST ǀ MRI1 ǀ MSMO1 ǀ MTHFD2 ǀ NAXE ǀ NCOR2 ǀ ND1 ǀ ND2 ǀ ND4 ǀ ND5 ǀ NDUFA1 ǀ NDUFA10 ǀ NDUFA12 ǀ NDUFA13 ǀ NDUFA2 ǀ NDUFA3 ǀ NDUFA4 ǀ NDUFA7 ǀ NDUFA9 ǀ NDUFAF2 ǀ NDUFB1 ǀ NDUFB10 ǀ NDUFB11 ǀ NDUFB3 ǀ NDUFB4 ǀ NDUFB5 ǀ NDUFB6 ǀ NDUFB7 ǀ NDUFB8 ǀ NDUFB9 ǀ NDUFC2 ǀ NDUFS1 ǀ NDUFS2 ǀ NDUFS5 ǀ NDUFS6 ǀ NDUFS7 ǀ NDUFS8 ǀ NDUFV1 ǀ NDUFV2 ǀ NFS1 ǀ NFYA ǀ NUDT1 ǀ NUDT19 ǀ NUP153 ǀ NUP58 ǀ NUP62 ǀ PAH ǀ PCBD1 ǀ PCK2 ǀ PCYT1A ǀ PCYT2 ǀ PDHB ǀ PGLS ǀ PIK3CB ǀ PIK3R2 ǀ PIP4P1 ǀ PIP5K1A ǀ PISD ǀ POLD1 ǀ PON2 ǀ PPCS ǀ PPP1CA ǀ PPP1CB ǀ PPP2CA ǀ PRSS3 ǀ PSMB4 ǀ PSMB7 ǀ PTDSS1 ǀ PXMP2 ǀ PYCR3 ǀ RAB4A ǀ RAB5A ǀ RAE1 ǀ RANBP2 ǀ RAP1A ǀ RDH11 ǀ RPL13 ǀ RPL14 ǀ RPL18 ǀ RPL22L1 ǀ RPL23A ǀ RPL24 ǀ RPL26L1 ǀ RPL29 ǀ RPL31 ǀ RPL32 ǀ RPL34 ǀ RPL35 ǀ RPL36A ǀ RPL6 ǀ RPL7A ǀ RPLP1 ǀ RPS24 ǀ RPS26 ǀ SACM1L ǀ SCO1 ǀ SDC4 ǀ SDHA ǀ SEC13 ǀ SERINC1 ǀ

	SGPP1 | SLC16A1 | SLC25A10 | SLC25A11 | SLC25A13 | SLC2A1 | SLC35B2 | SLC7A5 | SMPD4 | SMS | SPTLC2 | SQOR | SUCLA2 | SURF1 | THRAP3 | TIMMDC1 | TM7SF2 | TST | TXNRD1 | UQCR10 | UQCR11 | UQCRC1 | UQCRC2 | UQCRFS1 | UQCRQ | VDAC1
Metabolism of RNA	BCAS2 | BUD31 | CDC5L | CLNS1A | CPSF2 | CPSF3 | CPSF4 | CSNK1D | CTNNBL1 | DIMT1 | EBNA1BP2 | EIF4B | ERCC2 | EXOSC2 | EXOSC6 | EXOSC7 | FCF1 | FIP1L1 | FTSJ3 | GEMIN7 | GNL3 | GSPT2 | GTPBP3 | HNRNPH1 | HNRNPH2 | HNRNPR | HSD17B10 | IMP3 | IMP4 | KRR1 | LSM4 | MPHOSPH10 | MRM1 | MTO1 | NAT10 | NCBP2 | NHP2 | NOP10 | NOP2 | NOP58 | NUP153 | NUP58 | NUP62 | PABPN1 | PAPOLA | PELP1 | PHF5A | POLR2E | POLR2G | POLR2I | POLR2L | POP4 | PPIL4 | PPP2CA | PPWD1 | PRKCD | PRPF40A | PSMB4 | PSMB7 | RAE1 | RANBP2 | RIOK2 | RNPS1 | RPL13 | RPL14 | RPL18 | RPL22L1 | RPL23A | RPL24 | RPL26L1 | RPL29 | RPL31 | RPL32 | RPL34 | RPL35 | RPL36A | RPL6 | RPL7A | RPLP1 | RPP38 | RPP40 | RPS24 | RPS26 | RRP9 | SART1 | SEC13 | SLBP | SMNDC1 | SNRPC | SNU13 | SRRM1 | SRRM2 | SRSF3 | SUGP1 | THOC3 | TRMT5 | TRMT6 | TRMT61A | TSEN2 | TSEN34 | UPF3B | UTP20 | UTP3 | UTP6 | WBP11 | WDR61 | WDR75
Amino acid metabolism	ACAD8 | ALDH4A1 | ARG1 | AUH | AZIN2 | DLAT | DLST | GCDH | GOT1 | HSD17B10 | IVD | KYAT3 | MPST | MRI1 | PAH | PCBD1 | PDHB | PSMB4 | PSMB7 | PXMP2 | PYCR3 | RPL13 | RPL14 | RPL18 | RPL22L1 | RPL23A | RPL24 | RPL26L1 | RPL29 | RPL31 | RPL32 | RPL34 | RPL35 | RPL36A | RPL6 | RPL7A | RPLP1 | RPS24 | RPS26 | SERINC1 | SLC25A10 | SLC25A13 | SLC7A5 | SMS | SQOR | TST | TXNRD1
Metabolism of lipids	ABCC1 | ACACA | ACACB | ACADVL | ACAT2 | ACOT13 | ACSF3 | AGK | AGPAT5 | CDIPT | CDS2 | CERS2 | COL4A3BP | CTSA | DGAT1 | DHCR7 | DHRS7B | ELOVL1 | ELOVL5 | ELOVL7 | EP300 | ESYT2 | FABP5 | GPAT3 | GPD1L | HACD2 | HELZ2 | LBR | LCLAT1 | LPCAT1 | LPCAT3 | MBOAT7 | MCAT | ME1 | MED19 | MSMO1 | NCOR2 | NFYA | NUDT19 | PCYT1A | PCYT2 | PIK3CB | PIK3R2 | PIP4P1 | PIP5K1A | PISD | PON2 | PPP1CA | PPP1CB | PTDSS1 | RAB4A | RAB5A | SACM1L | SGPP1 | SMPD4 | SPTLC2 | THRAP3 | TM7SF2 | TXNRD1
Metabolism of proteins	ACADVL | ACTL6A | ATP6V0D1 | AURKAIP1 | B2M | B4GALT5 | BABAM2 | BET1L | BLM | CALM3 | CBX4 | CHCHD1 | CHD3 | CKAP4 | CMAS | COG3 | COPS7A | CREBRF | CSNK1D | CTR9 | CTSA | CUL4A | CUL5 | DAD1 | DCUN1D5 | DERL2 | DPM3 | DYNC1I2 | DYNLL2 | EIF1AX | EIF4B | EIF5B | EP300 | ETFB | EXOC4 | EXOSC2 | EXOSC6 | EXOSC7 | F10 | FBXO22 | FKBP8 | FOXK2 | GADD45GIP1 | GALNT2 | GAN | GFM2 | GMPPA | GNG12 | GPAA1 | GSPT2 | HARS2 | HDGF | HIST2H2AB | HIST2H2AC | KTN1 | LMAN2 | LMNA | MAGT1 | MAVS | MLEC | MPI | MRPL11 | MRPL13 | MRPL14 | MRPL15 | MRPL16 | MRPL17 | MRPL2 | MRPL21 | MRPL22 | MRPL23 | MRPL24 | MRPL27 | MRPL28 | MRPL30 | MRPL32 | MRPL34 | MRPL40 | MRPL41 | MRPL42 | MRPL43 | MRPL46 | MRPL48 | MRPL54 | MRPL55 | MRPL58 | MRPS10 | MRPS12 | MRPS15 | MRPS21 | MRPS23 | MRPS24 | MRPS25 | MRPS30 | MRPS31 | MRPS33 | MRPS34 | MRPS5 | MRRF | MTIF2 | MUCL1 | NARS2 | NCOR2 | NFYA | NOP58 | NUP153 | NUP58 | NUP62 | OGT | PFDN2 | PHC2 | PIGT | PIGU | PML | PPP6R3 | PSMB4 | PSMB7 | PTRH2 | RAB22A | RAB2B | RAB35 | RAB3A | RAB3B | RAB4A | RAB4B | RAB5A | RAB5B | RAB6A | RABGGTB | RAE1 | RANBP2 | RBBP7 | RBX1 | RHOT1 | RNF2 | RNF20 | RPL13 | RPL14 | RPL18 | RPL22L1 | RPL23A | RPL24 | RPL26L1 | RPL29 | RPL31 | RPL32 | RPL34 | RPL35 | RPL36A | RPL6 | RPL7A | RPLP1 | RPS24 | RPS26 | SARS2 | SEC11A | SEC13 | SEC22B | SEC61A1 | SEC61B | SMAD3 | SPCS2 | SPTBN1 | SRP19 | SRP72 | SSR1 | SSR3 | STAG2 | TGOLN2 | TMED10 | TOP1 | TRAM1 | TRRAP | TTF1 | TTLL11 | UBA3 | UBE2A | UBE2L6 | UBE2T | UBE2V2 | UCHL3 | USP16 | USP19 | USP42 | VDAC1 | VDAC2 | VDAC3 | WDR61 | WFS1 | XRCC4 | YIF1A
Neutrophil degranulation	ACTR1B | AHCYL1 | ANAPC7 | AP1S1 | AP2A2 | AP2M1 | ARG1 | ARPC1A | ARPC1B | ATP6V0A1 | ATP6V0D1 | ATP6V1E1 | ATP6V1G1 | B2M | BLMH | C1orf35 | CAB39 | CALM3 | CALML5 | CAMK2G | CAT | CD81 | CD99 | CDC27 | CDH1 | CEP290 | CHUK | CKAP4 | CREB1 | CRK | CRKL | CSK | CTSA | CTSB | CUL5 | CYB5R3 | CYBA | CYFIP2 | CYSTM1 | DCD | DGAT1 | DSC1 | DSG1 | DTX3L | DYNC1I2 | DYNLL2 | DYNLT1 | EP300 | EREG | FABP5 | FBXO22 | GAN | GSDMD | HBB | HERC4 | HLA-G | HSPA6 | IFITM3 | IL18 | ISG15 | IST1 | JUP | KCMF1 | KIF23 | LAMTOR3 | LPCAT1 | LRSAM1 | LYN | MAGT1 | MAVS | MGST1 | MIF | MLEC | MUCL1 | MYO1C | NDUFC2 | NEDD4 | NFKB1 | NRAS | NUP153 | NUP58 | NUP62 | OASL | OSTF1 | PDAP1 | PEA15 | PEBP1 | PGRMC1 | PHB | PIK3CB |

	PIK3R2 ǀ PKP1 ǀ PML ǀ POLR2E ǀ POLR2L ǀ POLR3C ǀ PPP2CA ǀ PPP5C ǀ PRDX4 ǀ PRKCD ǀ PRSS3 ǀ PSMB4 ǀ PSMB7 ǀ PTK2 ǀ PVR ǀ RAB3A ǀ RAB4B ǀ RAB5B ǀ RAB6A ǀ RAC1 ǀ RAC2 ǀ RACGAP1 ǀ RAE1 ǀ RALA ǀ RANBP2 ǀ RANBP9 ǀ RAP1A ǀ RAP2B ǀ RAP2C ǀ RBBP6 ǀ RBX1 ǀ RNF126 ǀ S100A8 ǀ S100A9 ǀ SCAMP1 ǀ SEC13 ǀ SEC22B ǀ SEC61A1 ǀ SEC61B ǀ SERPINB12 ǀ SMAD3 ǀ SNAP23 ǀ SNAP29 ǀ SPTBN1 ǀ SQSTM1 ǀ STOM ǀ STX3 ǀ TAB2 ǀ TAP2 ǀ TOLLIP ǀ TRIM21 ǀ TRIM56 ǀ UBA3 ǀ UBE2A ǀ UBE2L6 ǀ UBE2V2 ǀ UBE4A ǀ VIM
Respiratory electron transport	ACTL6A ǀ AKT1S1 ǀ AP2A2 ǀ AP2M1 ǀ ARID1B ǀ ATP5MG ǀ ATP5PF ǀ ATP6 ǀ ATP6V0A1 ǀ ATP6V0D1 ǀ ATP6V1E1 ǀ ATP6V1G1 ǀ BAD ǀ BSG ǀ CALM3 ǀ CALML5 ǀ COA3 ǀ COX1 ǀ COX17 ǀ COX2 ǀ COX20 ǀ COX4I1 ǀ COX5B ǀ COX6C ǀ COX7A2L ǀ CREB1 ǀ CYC1 ǀ DLAT ǀ DLST ǀ DNAH5 ǀ EP300 ǀ ETFB ǀ GAPDH ǀ HSD17B10 ǀ HTRA2 ǀ LHPP ǀ MAPT ǀ MDH2 ǀ ME1 ǀ MLXIPL ǀ ND1 ǀ ND2 ǀ ND4 ǀ ND5 ǀ NDUFA1 ǀ NDUFA10 ǀ NDUFA12 ǀ NDUFA13 ǀ NDUFA2 ǀ NDUFA3 ǀ NDUFA4 ǀ NDUFA7 ǀ NDUFA9 ǀ NDUFAF2 ǀ NDUFB1 ǀ NDUFB10 ǀ NDUFB11 ǀ NDUFB3 ǀ NDUFB4 ǀ NDUFB5 ǀ NDUFB6 ǀ NDUFB7 ǀ NDUFB8 ǀ NDUFB9 ǀ NDUFC2 ǀ NDUFS1 ǀ NDUFS2 ǀ NDUFS5 ǀ NDUFS6 ǀ NDUFS7 ǀ NDUFS8 ǀ NDUFV1 ǀ NDUFV2 ǀ NFKB1 ǀ NRAS ǀ PDHB ǀ PIK3CB ǀ PIK3R2 ǀ POLR2E ǀ POLR2G ǀ POLR2I ǀ POLR2L ǀ PRKAA1 ǀ RAC1 ǀ RPS6KB1 ǀ RPTOR ǀ RTN3 ǀ SCO1 ǀ SDHA ǀ SIRT6 ǀ SLC16A1 ǀ SLC25A4 ǀ SUCLA2 ǀ SURF1 ǀ TBP ǀ TIMMDC1 ǀ UBE2L6 ǀ UQCR10 ǀ UQCR11 ǀ UQCRC1 ǀ UQCRC2 ǀ UQCRFS1 ǀ UQCRQ ǀ VDAC1 ǀ VDAC2 ǀ VDAC3
Signaling by Receptor Tyrosine Kinases	AHCYL1 ǀ AP2A2 ǀ AP2M1 ǀ ATP6V0A1 ǀ ATP6V0D1 ǀ ATP6V1E1 ǀ ATP6V1G1 ǀ CALM3 ǀ CREB1 ǀ CRK ǀ CRKL ǀ CSK ǀ CUL5 ǀ CYBA ǀ CYFIP2 ǀ EPS15 ǀ EPS15L1 ǀ EREG ǀ GRB7 ǀ HNRNPH1 ǀ ITGA2 ǀ ITGA3 ǀ JUP ǀ LAMB3 ǀ LYN ǀ MEMO1 ǀ NCBP2 ǀ NEDD4 ǀ NRAS ǀ PIK3CB ǀ PIK3R2 ǀ POLR2E ǀ POLR2G ǀ POLR2I ǀ POLR2L ǀ PPP2CA ǀ PRKCD ǀ PTK2 ǀ RAB4A ǀ RAB4B ǀ RAC1 ǀ RALA ǀ RALB ǀ RANBP9 ǀ RAP1A ǀ SPINT1 ǀ TAB2
Transcriptional Regulation by TP53	ACTL6A ǀ AIFM2 ǀ ANAPC7 ǀ ARID1B ǀ ATRIP ǀ BAZ2A ǀ BLM ǀ BRD7 ǀ CALM3 ǀ CAMK2G ǀ CAT ǀ CBFB ǀ CBX3 ǀ CBX4 ǀ CCNT1 ǀ CDC27 ǀ CHD3 ǀ COX1 ǀ COX2 ǀ COX20 ǀ COX4I1 ǀ COX5B ǀ COX6C ǀ COX7A2L ǀ CPSF2 ǀ CPSF3 ǀ CPSF4 ǀ CREB1 ǀ CTR9 ǀ EHMT1 ǀ ELL ǀ EP300 ǀ ERCC2 ǀ FANCI ǀ FIP1L1 ǀ GTF2E2 ǀ GTF3C2 ǀ GTF3C4 ǀ GTF3C5 ǀ H2AFV ǀ HIST2H2AC ǀ INTS2 ǀ INTS9 ǀ IWS1 ǀ LAMTOR3 ǀ LBR ǀ MBD2 ǀ MECP2 ǀ MTA2 ǀ MTA3 ǀ MYO1C ǀ NCBP2 ǀ NCOR2 ǀ NDUFA4 ǀ NELFE ǀ NFKB1 ǀ NFYA ǀ NOP2 ǀ NUP153 ǀ NUP58 ǀ NUP62 ǀ OCLN ǀ PABPN1 ǀ PAPOLA ǀ PHC2 ǀ PIP4P1 ǀ PML ǀ POLR1A ǀ POLR1B ǀ POLR2E ǀ POLR2G ǀ POLR2I ǀ POLR2L ǀ POLR3C ǀ PPP2CA ǀ PRKAA1 ǀ PSMB4 ǀ PSMB7 ǀ RABGGTB ǀ RAE1 ǀ RANBP2 ǀ RBBP7 ǀ RBM14 ǀ RBPJ ǀ RBX1 ǀ RNF2 ǀ RNPS1 ǀ RPA2 ǀ RPA3 ǀ RPTOR ǀ SCO1 ǀ SEC13 ǀ SLBP ǀ SMAD3 ǀ SOX9 ǀ SRRM1 ǀ SRSF3 ǀ SSU72 ǀ SURF1 ǀ TAF2 ǀ TAF3 ǀ TBP ǀ TFAP2D ǀ THOC3 ǀ TMEM219 ǀ TNRC6B ǀ TPX2 ǀ TRIM33 ǀ TTF1 ǀ TXNRD1 ǀ UPF3B ǀ WDR61
Translation	AURKAIP1 ǀ CHCHD1 ǀ EIF1AX ǀ EIF4B ǀ EIF5B ǀ GADD45GIP1 ǀ GFM2 ǀ GSPT2 ǀ HARS2 ǀ MRPL11 ǀ MRPL13 ǀ MRPL14 ǀ MRPL15 ǀ MRPL16 ǀ MRPL17 ǀ MRPL2 ǀ MRPL21 ǀ MRPL22 ǀ MRPL23 ǀ MRPL24 ǀ MRPL27 ǀ MRPL28 ǀ MRPL30 ǀ MRPL32 ǀ MRPL34 ǀ MRPL40 ǀ MRPL41 ǀ MRPL42 ǀ MRPL43 ǀ MRPL46 ǀ MRPL48 ǀ MRPL54 ǀ MRPL55 ǀ MRPL58 ǀ MRPS10 ǀ MRPS12 ǀ MRPS15 ǀ MRPS21 ǀ MRPS23 ǀ MRPS24 ǀ MRPS25 ǀ MRPS30 ǀ MRPS31 ǀ MRPS33 ǀ MRPS34 ǀ MRPS5 ǀ MRRF ǀ MTIF2 ǀ NARS2 ǀ RPL13 ǀ RPL14 ǀ RPL18 ǀ RPL22L1 ǀ RPL23A ǀ RPL24 ǀ RPL26L1 ǀ RPL29 ǀ RPL31 ǀ RPL32 ǀ RPL34 ǀ RPL35 ǀ RPL36A ǀ RPL6 ǀ RPL7A ǀ RPLP1 ǀ RPS24 ǀ RPS26 ǀ RSL24D1 ǀ SARS2 ǀ SEC11A ǀ SEC61A1 ǀ SEC61B ǀ SPCS2 ǀ SRP19 ǀ SRP72 ǀ SSR1 ǀ SSR3 ǀ TRAM1
Vesicle-mediated transport	AP1S1 ǀ AP2A2 ǀ AP2M1 ǀ ARPC1A ǀ BET1L ǀ BNIP1 ǀ CALM3 ǀ CHMP2A ǀ CHMP4B ǀ COG3 ǀ COPS7A ǀ CSNK1D ǀ DYNC1I2 ǀ DYNLL2 ǀ EPS15 ǀ EPS15L1 ǀ EREG ǀ EXOC4 ǀ GAK ǀ GALNT2 ǀ GCC2 ǀ HBB ǀ HPX ǀ KIF18B ǀ KIF23 ǀ KIFC1 ǀ LMAN2 ǀ MVB12A ǀ MYO1C ǀ NECAP2 ǀ PPP6R3 ǀ RAB35 ǀ RAB3A ǀ RAB4A ǀ RAB5A ǀ RAB5B ǀ RAB6A ǀ RAC1 ǀ RACGAP1 ǀ RALA ǀ RIN1 ǀ SCARB1 ǀ SCARB2 ǀ SEC13 ǀ SEC22B ǀ SNAP23 ǀ SNAP29 ǀ SPTBN1 ǀ STX6 ǀ SYT1 ǀ TGOLN2 ǀ TMED10 ǀ TPD52 ǀ VPS25 ǀ VTA1 ǀ YIPF6
